# 2297. COVID-19 Vaccine Effectiveness Among Pregnant People

**DOI:** 10.1093/ofid/ofac492.135

**Published:** 2022-12-15

**Authors:** Ousseny Zerbo, G Thomas Ray, Kristin Goddard, Evan Layefsky, Bruce Fireman, Nicola P Klein

**Affiliations:** Division of Research Kaiser Permanente Vaccine Study Center, Oakland, California; Division of Research Kaiser Permanente Vaccine Study Center, Oakland, California; Division of Research Kaiser Permanente Vaccine Study Center, Oakland, California; Division of Research Kaiser Permanente Vaccine Study Center, Oakland, California; Division of Research Kaiser Permanente Vaccine Study Center, Oakland, California; Division of Research Kaiser Permanente Vaccine Study Center, Oakland, California

## Abstract

**Background:**

COVID-19 presents a serious health risk to pregnant people and pregnancy outcomes. However, pregnant people were not included in pivotal phase III COVID-19 vaccine efficacy trials.

**Methods:**

We used Cox regression models in a cohort study to determine hazard ratios (HR) of a PCR positive test (“infection”) comparing vaccinated with unvaccinated pregnant persons in Kaiser Permanente Northern California. HRs were adjusted for age, race/ethnicity, type of insurance coverage, geographical area, BMI, preexisting diabetes, hypertension, parity, time since pregnancy onset and smoking status. Vaccine effectiveness (VE), calculated as 1 minus adjusted HR, was estimated for fully vaccinated < 150 and ≥ 150 days prior to infection. VE was estimated for before and during Delta, and Omicron. We also calculated incidence rates of COVID-pneumonia associated hospitalization by vaccination status.

**Results:**

Among 68836 pregnancies between 12/15/2020 and 3/31/2022, 21834 (31.7%) were fully vaccinated and 5980 (8.7%) were boosted by the end of pregnancy. Compared with unvaccinated persons, the HRs of infection for fully vaccinated < 150 days prior were 0.13 (95% CI: 0.07 – 0.23; VE=87% [77% - 93%]) before Delta; 0.25 (CI: 0.20 – 0.30; VE=75% [70% - 80%]) during Delta and 0.76 (CI: 0.61 – 0.94; VE= 24% [16% - 39%]) during Omicron. The HRs for ≥ 150 days prior were 0.38 (CI: 0.31 – 0.46; VE=62 % [54% - 69%]) during Delta and 1.04 (CI: 0.89 – 1.22; VE= -0.04% [-0.22% – 0.11%]) during Omicron. The HRs for boosted persons were 0.10 (CI: 0.04 – 0.25; VE= 90% [75% - 96%]) during Delta and 0.42 (CI: 0.34 – 0.52; VE=58% [48% - 66%]) during Omicron periods.

Incidence rates (IR) per 1000 person-years for hospitalization before delta were 0.75 among unvaccinated and zero among vaccinated. During Delta, the IR was 6.64 for unvaccinated and zero for fully vaccinated and boosted. During Omicron, the IR was 10.27 for unvaccinated, zero for fully vaccinated < 150 days prior, 2.48 for fully vaccinated ≥ 150 days prior and zero for those boosted.

**Conclusion:**

COVID-19 vaccines protect against infection and hospitalization among pregnant people. However, vaccine effectiveness against infection wanes over time and was lower during Omicron. Booster doses are necessary for continuous protection.

**Disclosures:**

**All Authors**: No reported disclosures.

